# Understanding the Mechanism of Antidepressant-Related Sexual Dysfunction: Inhibition of Tyrosine Hydroxylase in Dopaminergic Neurons after Treatment with Paroxetine but Not with Agomelatine in Male Rats

**DOI:** 10.3390/jcm8020133

**Published:** 2019-01-23

**Authors:** Yanira Santana, Angel L. Montejo, Javier Martín, Ginés LLorca, Gloria Bueno, Juan Luis Blázquez

**Affiliations:** 1 Department of Psychiatry, Hospital Universitario de Salamanca, 37007 Salamanca, Spain; doctorayani@hotmail.com; 2 University of Salamanca, IBSAL, Nursing School E.U.E.F., 37007 Salamanca, Spain; 3 Department of Statistics, School of Medicine, University of Salamanca, 37007 Salamanca, Spain; jmv@usal.es; 4 Department of Psychiatry, School of Medicine, University of Salamanca, 37007 Salamanca, Spain; gllorca@usal.es (G.L.); gloriabueno@usal.es (G.B.); 5 Department of Human Anatomy and Histology, IBSAL NEUR-2, School of Medicine, University of Salamanca, 37007 Salamanca, Spain; jlba@usal.es

**Keywords:** dopaminergic system, paroxetine, agomelatine, immunohistochemical study, sexual dysfunction, male rats

## Abstract

Antidepressant-related sexual dysfunction is a frequent adverse event caused by serotonergic activation that intensely affects quality of life and adherence in depressed patients. The dopamine system has multiple effects promoting sexual behavior, but no studies have been carried out to confirm dopaminergic changes involved in animal models after antidepressant use. Methods: The sexual behavior-related dopaminergic system in the rat was studied by comparing two different antidepressants and placebo for 28 days. The antidepressants used were paroxetine (a serotonergic antidepressant that causes highly frequent sexual dysfunction in humans) and agomelatine (a non-serotonergic antidepressant without associated sexual dysfunction). The tyrosine hydroxylase immunoreactivity (THI) in the substantia nigra pars compacta, the ventral tegmental area, the zona incerta, and the hypothalamic arcuate nucleus, as well as the dopaminergic projections to the striatum, hippocampus, cortex, and median eminence were analyzed. Results: The THI decreased significantly in the substantia nigra and ventral tegmental area after treatment with paroxetine, and the labeling was reduced drastically in the zona incerta and mediobasal hypothalamus. The immunoreactive axons in the target regions (striatum, cortex, hippocampus, and median eminence) almost disappeared only in the paroxetine-treated rats. Conversely, after treatment with agomelatine, a moderate reduction in immunoreactivity in the substantia nigra was found without appreciable modifications in the ventral tegmental area, zona incerta, and mediobasal hypothalamus. Nevertheless, no sexual or copulatory behavior was observed in any of the experimental or control groups. Conclusion: Paroxetine but not agomelatine was associated with important decreased activity in dopaminergic areas such as the substantia nigra and ventral tegmental areas that could be associated with sexual performance impairment in humans after antidepressant treatment.

## 1. Introduction

The dopaminergic (DA) and serotonergic (5-HT) modulatory systems are involved in regulating multiple functions through their abundant projections throughout the Central Nervous System (CNS). These systems are closely related and interact to control motor, cognitive, and affective functions. Dysfunction of these systems results in pathologies as marked as Parkinson’s disease, schizophrenia, depressive disorders, and Attention Deficit Hyperactivity Disorder (ADHD) AHDH syndrome.

The dopaminergic neurons are an anatomically and functionally heterogeneous group of cells, located in particular in the diencephalon and mesencephalon. In the murine brain, DA neurons are identified mainly in three structures. The first structure comprises the meso-diencephalic tegmental cell groups (A8–A10). Because these neurons originate from the substantia nigra (SN) and the ventral tegmental area (VTA) of the mesencephalon and diencephalon, we will refer to these neurons as meso-diencephalic dopaminergic (mdDA) neurons. They constitute the largest group of neurons and project to the striatum (nigrostriatal pathway), the limbic system (meso-limbic pathway), and the cerebral cortex (mesocortical pathway). The second structure is the zona incerta cell group (A13) in the ventral thalamus. The third structure comprises the hypothalamic (A12, A14, and A15) cell groups. The A12 group is the largest and provides the tuberoinfundibular and the tuberohypophysial projections involved in neuroendocrine regulation [1,2]. 

The interaction between DA and 5-HT systems is complex because it involves many types of membrane receptors that have mixed effects. The 5-HT neurons from the raphe nuclei send projections to dopaminergic cells in both the VTA and the SN, and to their terminals in the nucleus accumbens, prefrontal cortex, and striatum [3]. Some experimental data demonstrates that several 5-HT receptors subtypes (1a, 1b, 2a, 3, and 4) act to facilitate neuronal DA function and release, while the 5-HT2c receptor mediates an inhibitory effect on DA neuron activity and on DA release [4,5,6]. 

In recent years, antidepressant use has increased rapidly in Western countries because it is widely prescribed by psychiatrists and general practitioners. The introduction of selective serotonin reuptake inhibitors (SSRIs) in the late 1980s facilitated this process because of the alleged safety of these drugs compared with more dangerous drugs that were used previously [7,8]. However, some adverse effects of SSRIs are frequent and underestimated. One example is sexual dysfunction, which affects patients’ quality of life and continuity of treatment [9,10,11,12,13]. The incidence of sexual dysfunction is high (50–70%) when the mechanism of action is blocking serotonin reuptake, whereas drugs that act preferably on noradrenaline or dopamine reuptake have a less negative impact on the sexual function [14,15,16].

A high frequency of treatment discontinuation, close to 40%, has been notified in patients with major depression due to poor tolerance to antidepressant-related sexual dysfunction [15]. Several methods have been described for the therapeutic approach of this adverse event, including dose reduction, change to another antidepressant, or the use of corrective medication; unfortunately, none of these methods is completely effective deteriorating the quality of life of the patient in the long term [16,17].

Although mechanisms that cause sexual dysfunction still are not well understood, a recent study in rats suggests that the inhibitory effects of serotonergic antidepressants are related to the inhibitory effect of serotonin on dopamine release in hypothalamic and mesolimbic areas [18,19].

The inhibitory effect of serotonin on dopaminergic transmission was first shown by a reduction in nigral neuronal activity in response to electrical stimulation of the medial and dorsal raphe nucleus [20,21]. The increase in synaptic serotonin in response to SSRIs could then conceivably result in an amplification of the tonic inhibitory effects of serotonin, thereby leading to a reduction in DA transmission in the striatum [22]. This is supported by recent studies that have demonstrated a reduction in the substantia nigra tyrosine hydroxylase (TH) immunoreactive cell counts in response to SSRI administration [23]. 

SSRIs can also inhibit the basal activity of DA neurons in the VTA, which is strongly implicated in sexual desire and motivation. Thus, fluoxetine causes a dose-dependent inhibition of the VTA dopaminergic neuron firing rate, but it does not affect the activity of DA cells in other regions [24]. Acute injection of fluvoxamine, paroxetine, and sertraline produces a dose-dependent inhibition of some VTA DA neurons, but it does not affect the basal firing rate of other DA cells.

Agomelatine is a novel antidepressant drug that works on melatonergic (MT1 and MT2), and serotonergic (5-HT 2B and 5-HT2C) receptors [25]. Agomelatine has been used in two randomized studies in healthy male volunteers. These studies showed that agomelatine 25–50 mg/day is similar to placebo in sexual response, showing a lack of sexual dysfunction, whereas paroxetine 20 mg/day is related to a high sexual dysfunction frequency (>80% of patients showed decreased libido and orgasm delay) [26,27]. 

Our aim in this research is to study the dopaminergic system in male Wistar rats, especially the nuclei where neurons are located in the brainstem (substantia nigra pars compacta (SNc) and VTA), diencephalon (zona incerta (ZI) and hypothalamic arcuate nucleus (Arc)), and their most relevant axonal projections (striatum, hippocampus, hypothalamus, and cortex) in animals treated with paroxetine or agomelatine, which represents two different mechanisms of antidepressant action related to sexual adverse events. We will compare their effects on immunoreactivity to tyrosine hydroxylase, the rate-limiting enzyme of dopamine synthesis. The presence of this enzyme is considered a good marker of dopaminergic neurons in the central nervous system. We hypothesize that if the dopaminergic system is involved in sexual dysfunction caused by SSRIs, different antidepressant treatments will differentially modify TH immunoreactivity.

## 2. Material and Methods

Male Wistar rats, aged approximately 3 months old, were used. Rats were maintained under a 12 h light/dark cycle and at a constant temperature (20 °C) with free access to food and water. All animals were handled and cared for in accordance with the recommendations of European Commission and Spanish laws (2007/526/EC and RD 1201/2005). Authorization was requested to the Bioethics Committee of the University of Salamanca.

Twenty animals were distributed into the following groups: (1) four normal rats; (2) four rats treated orally with 10% hydroxyl-methyl-cellulose (the vehicle in which agomelatine was dissolved); (3) six rats treated orally with 10 mg/kg/day of paroxetine diluted in aqueous solution; and (4) six rats treated orally with 10 mg/kg/day of agomelatine diluted in 10% hydroxyl-methyl-cellulose. Because agomelatine needs to be dissolved in 10% hydroxyl-methyl-cellulose for absorption, an agomelatine control group was created with four rats that received only 10% hydroxyl-methyl-cellulose to observe its effects on the dopaminergic system. Since no differences were observed with the normal group, both finally were grouped as an only control group with eight rats.

Agomelatine solution was kindly provided by the manufacturer (Servier Lab) and paroxetine was obtained from the pharmacy. All treatments were performed for 28 days at 18:00 h each day. The size of the sample was empirically chosen due to the lack of previous evidence in the scientific literature on this topic.

Twenty-four hours after the end of treatment, rats were euthanized between 10:00 and 13:00 h. The brain was quickly extracted, the front and rear ends of the brain, the brainstem (pons and medulla oblongata), and the cerebellum were removed, and the remaining block was divided into two halves and fixed by immersion in Bouin’s fluid. Tissue was embedded in paraffin. The brain block was oriented to obtain coronal sections (8 μm thick). The whole block of tissue from each animal was serially cut and mounted (two sections per slide). Every tenth slide was stained with hematoxylin–eosin for orientation.

In order to observe the copulatory behavior and the possible differences between the experimental and control groups, two Wistar female rats were used. After 28 days, when the period of administration of paroxetine, agomelatine or placebo ended, one of the females was coupled in a new cage with one male from each group successively. Any sexual or approaching behavior was observed for a maximum of 5 min at 18:00 h, once for each male.

### 2.1. Immunohistochemistry

Selected sections were processed for tyrosine hydroxylase immunohistochemistry using the streptavidin–biotin method (EnVision, Dako, Denmark) with diaminobenzidine (DAB) as the electron donor. The antiserum (anti-tyrosine hydroxylase, GeneTex) was diluted in Tris buffer, pH 7.6, containing 0.7% non-gelling seaweed lambda carrageenan (Sigma) and 0.5% Triton X-100 (Sigma). The antiserum was used at a dilution of 1:300. The conditions and duration of incubation with the various reagents, especially with DAB and H_2_O_2_, was the same in all cases. Use of preimmune serum and omission of incubation in the primary antiserum during the immunostaining procedure were used as test controls and resulted in no immunostaining.

### 2.2. Quantification of Immunohistochemical Staining Intensity

Quantification of immunohistochemical staining intensity was performed using the open source software ImageJ (National Institutes of Health). We determined the pixel intensity of 60 immunoreactive neurons in the normal/control and experimental groups (we selected 15 neurons from the VTA nucleus and 15 from the SNc nucleus in four rats from each groups). To avoid possible differences in the pixel intensity resulting from the presence or absence of a cell nucleus, all measured cells had a visible cell nucleus. To determine the intensity of pixels, ImageJ assigns a value of 0 to the color black and a value of 255 to the color white. Thus, a greater staining intensity corresponds to a lower pixel intensity value.

### 2.3. Statistical Analysis

A two-factor ANOVA was used to analyze the differences in pixel intensity between groups and nuclei (SNc and VTA) and interaction between both factors. If the interaction between factors was statistically significant, one-way ANOVA was used to detect the differences between experimental groups for each level of area followed by Tukey’s multiple comparison tests where appropriate. Statistical significance was defined as *p* < 0.05. The mean and 95% confidence intervals (CIs) for each outcome are presented. Statistical analysis was conducted using the IBM SPSS 23 package (IBM, Armonk, NY, USA).

## 3. Results

In this study, images of the caudate-putamen, nucleus accumbens, and cortex were obtained from sections that correspond approximately with the coronal sections marked as Bregma 1.68–0.72 mm in the Paxinos and Watson atlas of the rat brain. Images of the zona incerta, arcuate nucleus and hippocampus correspond approximately with the coronal planes marked as Bregma −2.04 to −3.24 mm in the same atlas, and images of the VTA and SNc were obtained from sections that correspond approximately with the coronal sections marked as Bregma −4.80 to −5.28 mm in the rat brain atlas. The cortex images refer to the areas S1 (primary somatosensory cortex) and M1 (primary motor cortex) in the same atlas [27,28].

We found no differences in the staining intensity between normal and control rats treated with hydroxy-methyl-cellulose. Therefore, the findings in animals treated with paroxetine and agomelatine were analyzed in relation to the normal/control group of rats.

### 3.1. Substantia Nigra Compacta and the Ventral Tegmental Area

To identify the nuclei in DA neurons, we used the usual anatomical terms, and also referred to its name in the aminergic classification system by Dahlström and Fuxe (1964), in which the DA system is distributed into the groups A8–A14. 

**Figure 1:**
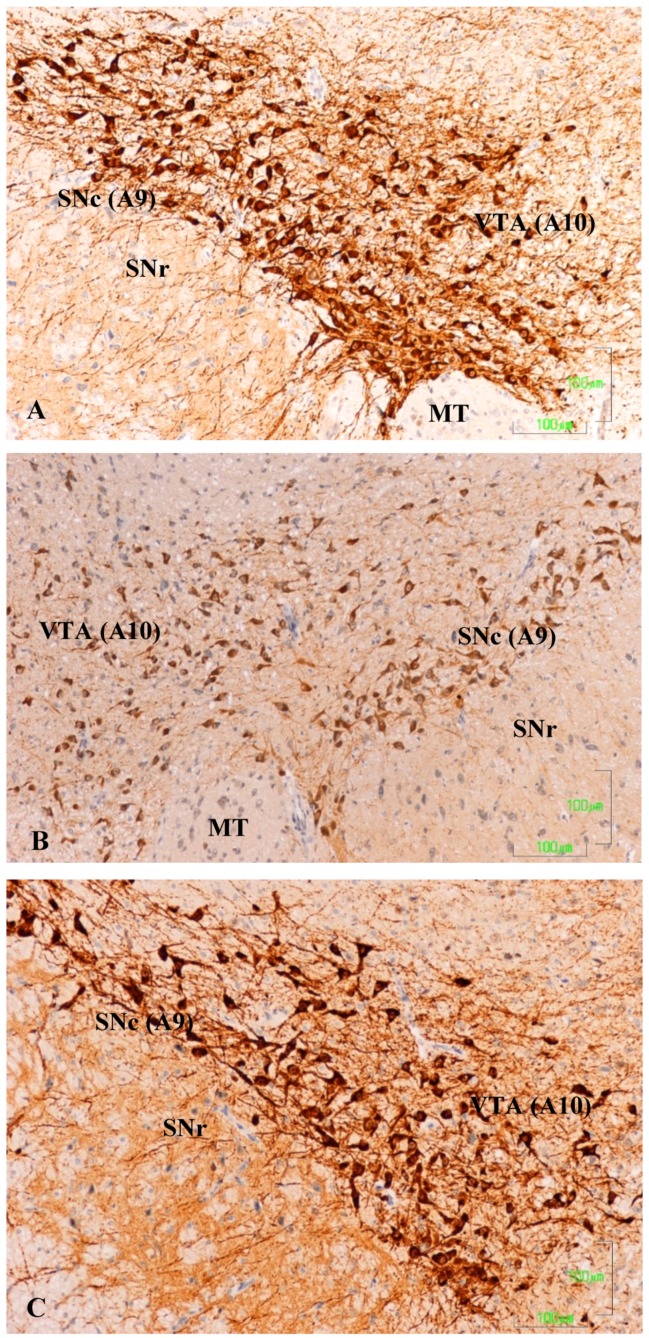
(**A**–**C**). Tyrosine hydroxylase immunoreactivity in the meso-diencephalic dopaminergic system of rats from the control (**A**), paroxetine (**B**), and agomelatine (**C**) groups. Bars, 100 m. SNc, substantia nigra pars compacta; SNr, substantia nigra pars reticulate; VTA, ventral tegmental area; MT, mammilothalamic tract.

In the substantia nigra (A9) and ventral tegmental area (A10) of control rats, TH neurons and neuronal processes are strongly reactive. Neuron labeling is intense throughout the cytoplasm, so that when the section affects the neuronal nucleus, it appears as a negative zone. TH axons surrounding the A9 and A10 nuclei also show a strong reaction, both penetrating the reticular substantia nigra (SNR) as located dorsally (Figure 1A). Conversely, in animals treated with paroxetine, the labeling is weak in both neurons and neuronal processes of the SNc and VTA nuclei. In the areas surrounding the nuclei, cited axons are barely visible (Figure 1B). In the SNc and VTA of agomelatine-treated rats, TH reactivity is similar to that described in the control rats, although labeling seems somewhat less intense (Figure 1C).

### 3.2. Striatum and Nucleus Accumbens 

**Figure 2:**
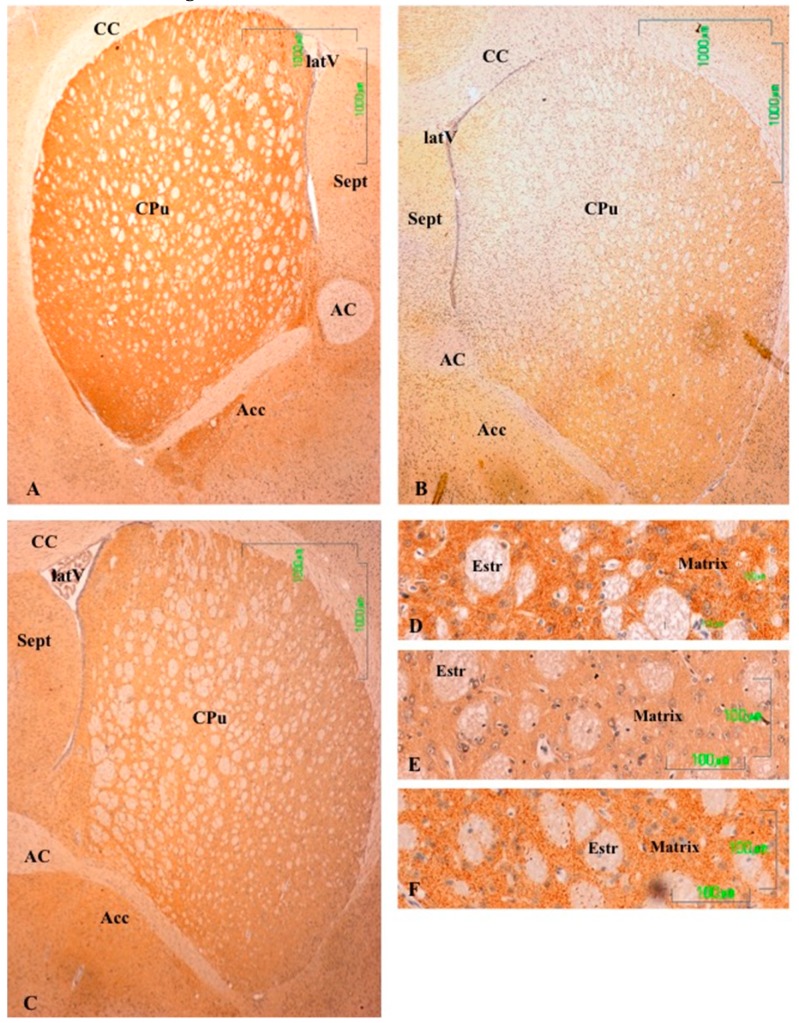
(**A**–**F**). Tyrosine hydroxylase immunoreactivity in the striatum of control rats (**A,D**), and rats treated with paroxetine (**B,E**) and agomelatine (**C,F**). Bars 1000 m (**A**–**C**) and 100 m (**D**–**F**). The reactivity is visible as dotted labeling that is evenly distributed by the matrix of the caudate-putamen (CPu), except in paroxetine-treated rats. CC, corpus callosum; latV, lateral ventriculum; AC, anterior commissure; Acc, nucleus accumbens; Estr, striatum; Sept, septum.

In the striatum (CPu) of the control animals, the labeling is intense and uniform throughout the matrix, but striosomes are negative (Figure 2A). In the nucleus accumbens, dopaminergic fibers are preferentially located in the lateral region. At higher magnifications, labeling is shown as dense dots, corresponding to the axons of the nigrostriatal pathway. We have not seen cell bodies of dopaminergic neurons in this region (Figure 2D).

The dopaminergic projections to the striatum are significantly affected after treatment with paroxetine, with the absence of immunoreactivity throughout the dorsal and medial CPu and significantly reduced immunoreactivity in the remaining area (Figure 2B). At higher magnifications, dopaminergic fibers have mostly disappeared (Figure 2E). In the striatum of animals treated with agomelatine, TH reactivity is reduced compared to the control group, but is more intense than in rats treated with paroxetine (Figure 2C,F). In the nucleus accumbens, the pattern of labeling is uniform throughout the CPu matrix (Figure 2C). 

### 3.3. Hippocampus

**Figure 3:**
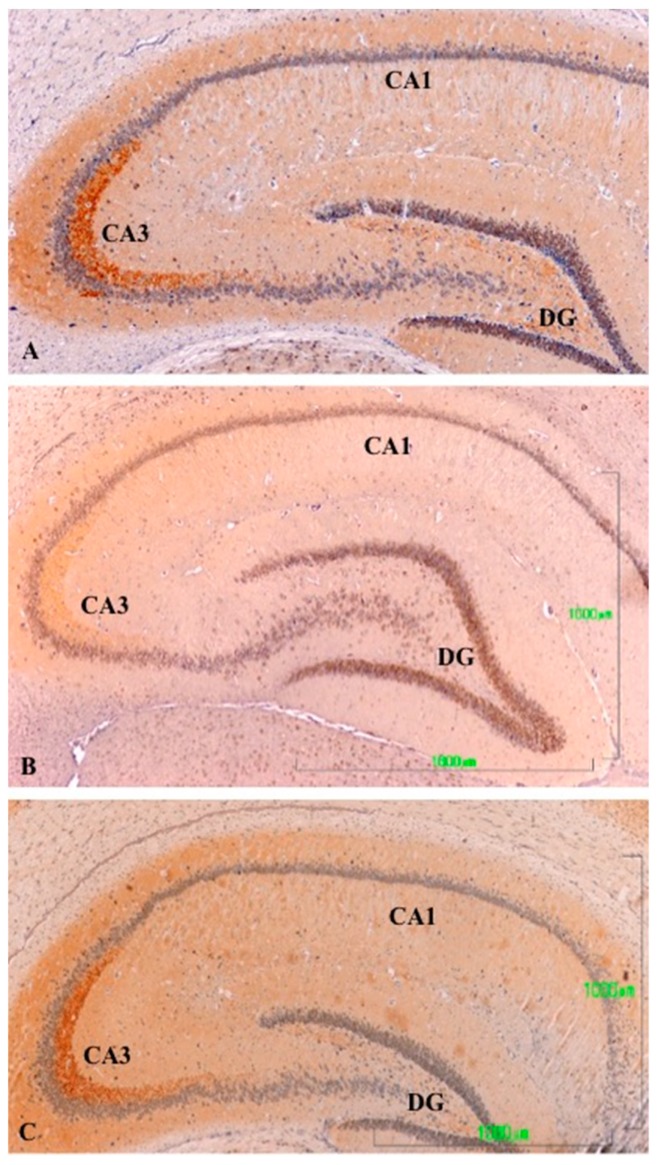
(**A**–**C**). Tyrosine hydroxylase immunoreactivity in the hippocampus of rats from the control (**A**), paroxetine (**B**), and agomelatine (**C**) groups. Bars, 1000 m. (Figure 3A is presented at the same magnification as that of Figure 3B,C). The immunoreactivity is limited to the CA3 area and is greatly reduced following treatment with paroxetine. DG, dentate gyrus; CA, cornu ammonis.

In the hippocampus of control animals, there was a strong reaction in the A3 region of the cornu ammonis (CA3; Figure 3A), which was shown by discrete labeling throughout the hippocampal region (Figure 3A lacks a bar, but the magnification is the same as in Figure 3B,C). After treatment with paroxetine, labeling of TH axons was strongly reduced, especially in CA3 (Figure 3B). Immunoreactivity slightly decreased in the agomelatine-treated group, even though reactive axons are observed (Figure 3C). We also detected TH-positive axons in the septum of rats in the control and agomelatine-treated groups, while labeling had also disappeared in paroxetine-treated animals.

### 3.4. Cerebral Cortex

**Figure 4:**
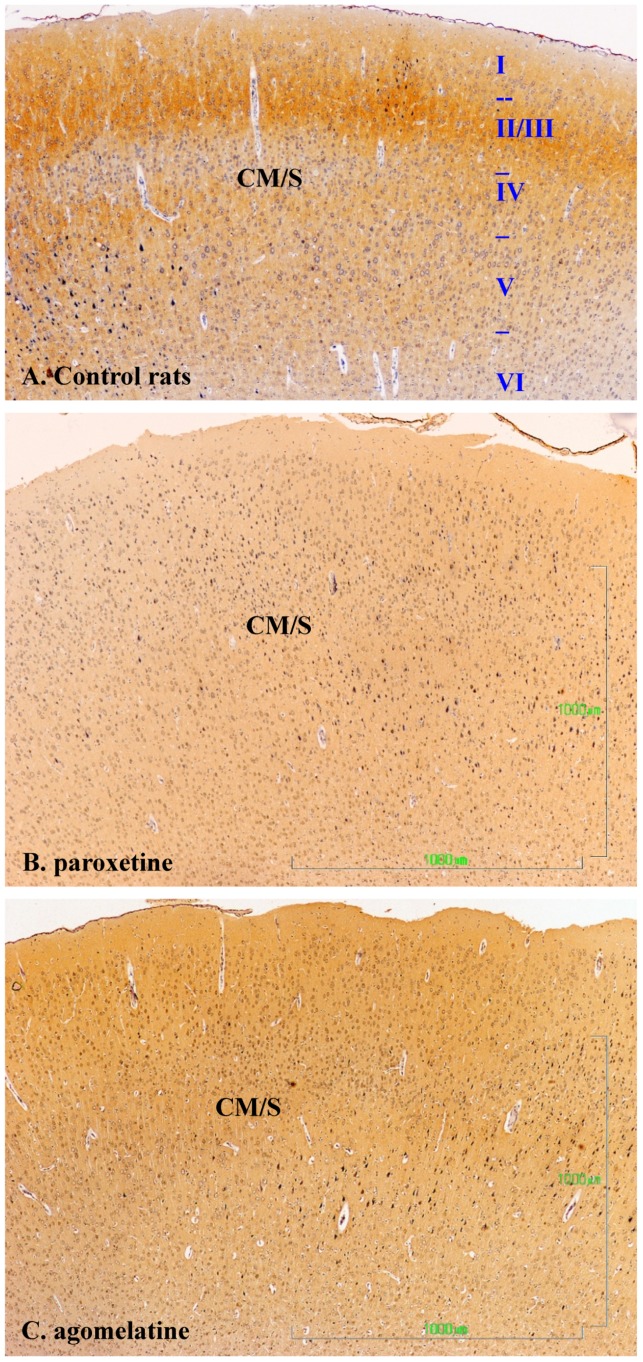
(**A**–**C**). Tyrosine hydroxylase immunoreactivity in the cerebral cortex layers I-VI of rats from the control (**A**), paroxetine (**B**), and agomelatine (**C**) groups. Bars, 1000 m. (Figure 4A is presented at the same magnification as that of Figure 4B,C). CM/S, motor/somatosensory cortex.

Figure 4 summarizes our observations on the dopaminergic innervation of the rat primary motor/somatosensory cortex (CM/S). This represents the mesocortical pathway. The reactivity in control rats is limited to axons and is located preferably in layers II/III, and it decreases both beneath and towards the surface (Figure 4A). Similar to other locations in rats treated with paroxetine, the labeling almost completely disappears (Figure 4B), but it is reduced in animals treated with agomelatine (Figure 4C).

### 3.5. Zona Incerta and Hypothalamus

**Figure 5:**
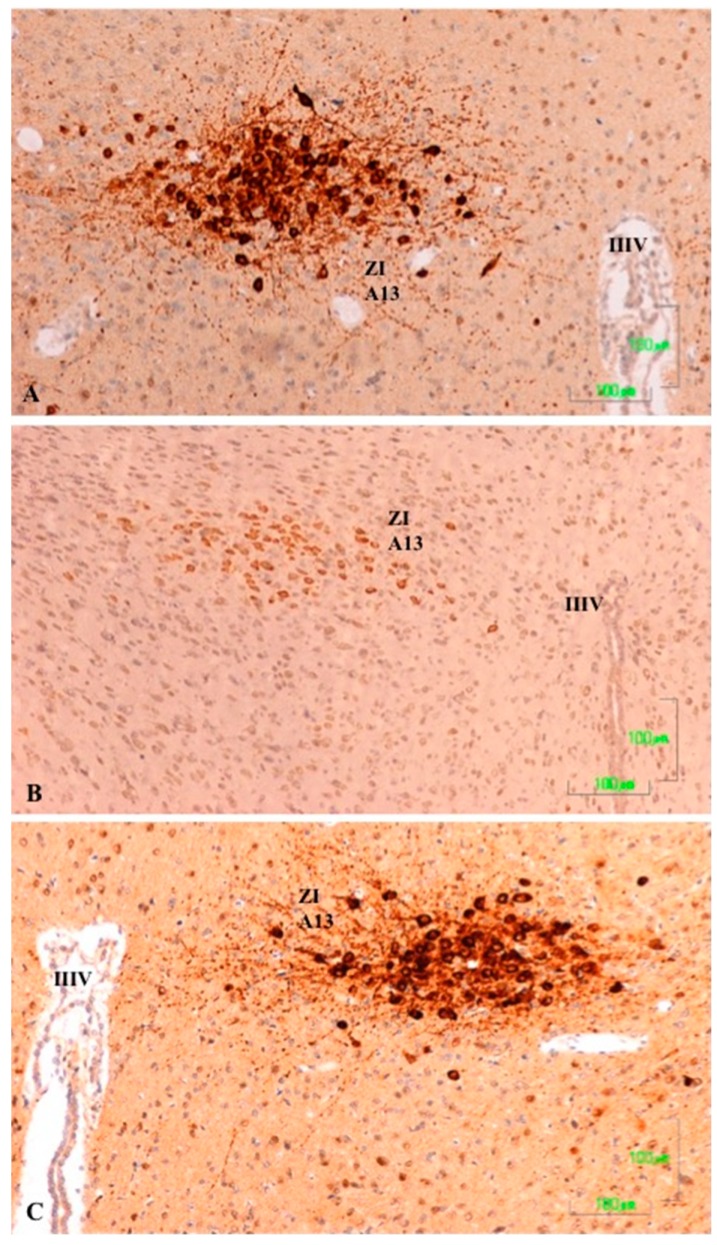
(**A**–**C**). Tyrosine hydroxylase immunoreactivity in the zona incerta of rats from the control (**A**), paroxetine (**B**), and agomelatine (**C**) groups. Bars, 100 m. In the rats treated with paroxetine, the labeling is weak whereas in animals treated with agomelatine, labeling is similar to that shown the control rats. IIIV, third ventricle; ZI, zona incerta.

In the control rats, TH-positive neurons in the zona incerta (A13) and fibers in and around the nucleus show intense labeling (Figure 5A). In rats treated with agomelatine, the appearance and reactivity of dopaminergic neurons is similar to that observed in the control group (Figure 5C). Conversely, in the animals treated with paroxetine, TH immunostaining is low in the neuronal cell bodies and it disappears in the nerve fibers (Figure 5B).

**Figure 6:**
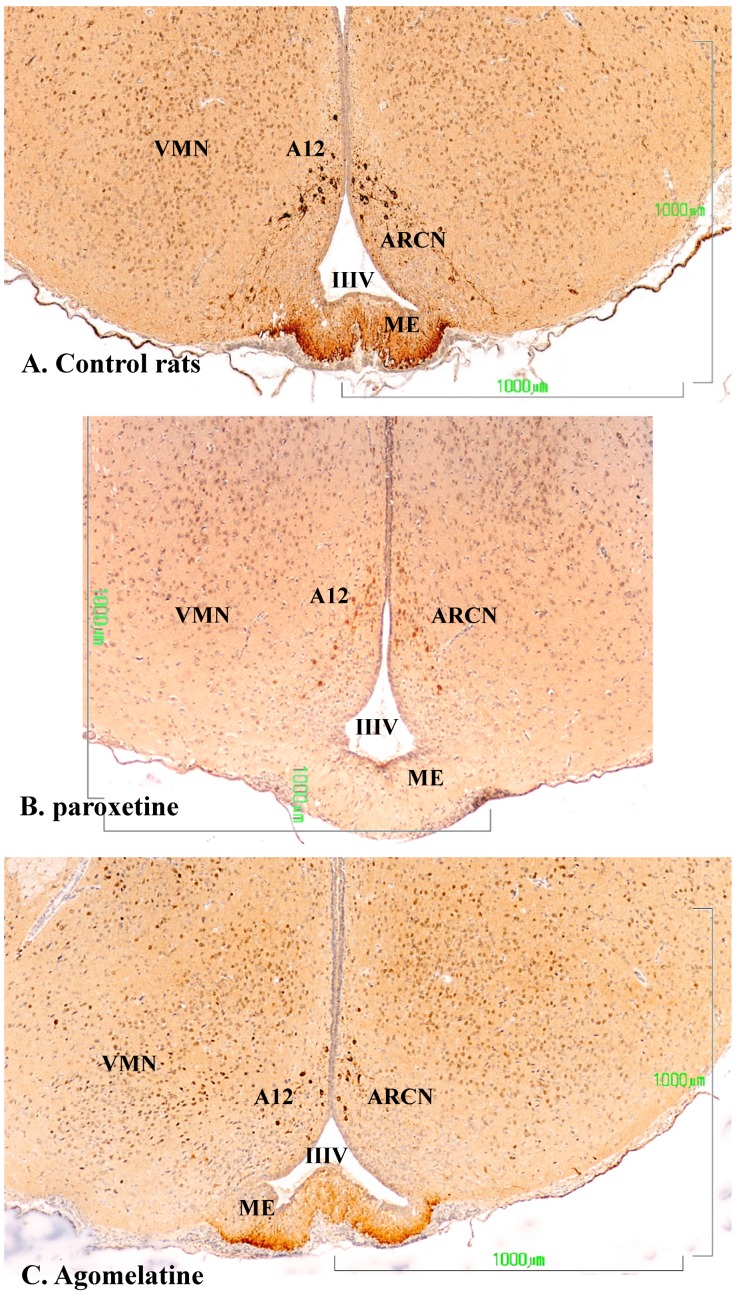
(**A**–**C**). Tyrosine hydroxylase immunoreactivity in the mediobasal hypothalamus of rats from the control (**A**), paroxetine (**B**), and agomelatine (**C**) groups. Bars, 1000 m. After treatment with paroxetine, TH immunoreactivity is absent from the median eminence. ARCN, arcuate nucleus; ME, median eminence; IIIV, third ventricle; VMN, ventromedial nucleus.

In the dopaminergic A12 group in the arcuate nucleus, the hypothalamic neurons behave similarly to those of the zona incerta (Figure 6). In the control group, the neurons show a more intense staining in the cell bodies of the arcuate nucleus (NARC) and in axons in the tuberoinfundibular tract that reach the outer zone of the median eminence (EM) (Figure 6A). Conversely, in the paroxetine-treated group, the arcuate neurons exhibit weak immunoreactivity, and the fibers of the median eminence are nonreactive (Figure 6B). In animals treated with agomelatine, the immunostaining is intense both in the neuronal bodies and in the median eminence, but it is somewhat reduced compared to control rats (Figure 6C).

### 3.6. Quantification of TH Immunoreactivity in the SNc and VTA 

**Figure 7:**
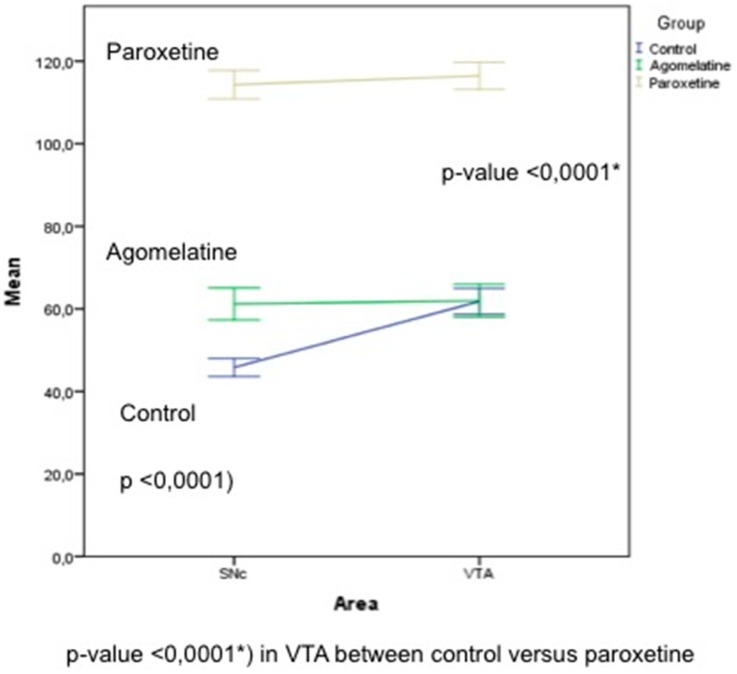
Pixel intensity was determined using open source software ImageJ. There are significant differences between all groups in the SNc, but no statistical difference between the control group and rats treated with agomelatine in the VTA.

As noted in the Materials and methods section, we used the open source software ImageJ to determine the pixel intensity in SNc and VTA neurons. This software assigns a value of 0 to the color black and a value of 255 to the color white. Thus, a greater staining intensity corresponds to a lower pixel value intensity. However, interaction between the experimental group and the area was detected (*p*-value < 0.0001, Figure 7). To analyze the interacction, we compared the differences between experimental groups by each area. There were statistical differences between the three experimental groups (*p* < 0.0001) in SNc, and between control and paroxetine groups in the VTA (*p* = 0.001).

### 3.7. Observation of Sexual and Mating Behaviour

The possible sexual or approaching behavior was observed for a maximum of 5 min, at 18:00 h. just once for each male and female couple. It was observed that the female rats were receptive for riding when meeting the male. However, the males in both experimental and control groups behaved sexually indifferent, showing stereotyped behaviors such as running around the cage and raising on their hind legs. No animals performed coitus with the female; therefore, we cannot draw any conclusions from this observation.

## 4. Discussion

To explain sexual dysfunction caused by treatment with SSRIs, various mechanisms have been proposed, among which is the inhibition of the dopaminergic system [18].

Given that the antidepressant-induced effects on sexual parameters in Wistar rats correspond well with their known effects in humans [14,15,29], we conducted a comparative experimental study on the dopaminergic system in male rats treated with paroxetine and agomelatine. This research improves our understanding of the mechanisms that explain sexual dysfunction, focusing on the meso-diencephalic dopaminergic system. 

Recently, MacGillivray et al. (2011) examined the effects of two different SSRIs, citalopram and fluoxetine, on cells containing tyrosine hydroxylase (TH) in nigrostriatal dopamine neurons and showed that both antidepressants induced a significant reduction in the number of TH cells in the Substantia Nigra. Our experimental model includes DA neurons from the substantia nigra and the VTA, and DA neurons of the zona incerta (ZI) and the tuberoinfundibular system. In this research, we did not count the immunoreactive TH cells, but we determined the intensity of staining in SNc and VTA TH cells. 

TH is the rate-limiting enzyme of dopamine synthesis, and it is considered one of the major agents in determining dopamine levels. When need for neurotransmitter increases at a DA synapse, TH is activated to make more DOPA. TH activity must be sustained until the need reduces and its activity must be turned off when the need for neurotransmitters has passed [30].

We believe it is relevant to clarify the meaning of changes in TH immunoreactivity regarding the available dopamine and dopaminergic system activity. What we show by immunohistochemical staining is the approximate number of immunoreactive TH molecules (the rate-limiting enzymes of catecholamine synthesis) in the areas studied, which is a good marker of dopamine neurons and fibers. Thus, under controlled staining conditions such as those in this study, more intense labeling means more TH molecules, which leads to an increased dopamine synthesis rate. Conversely, a reduction in the intensity of the labeling means generally fewer TH molecules and a decrease in DA synthesis. 

If we consider the large number of known 5-HT and DA receptors and the many factors that can influence regulation of sexual behavior, it is almost impossible to draw accurate conclusions. However, our data can complement those obtained using other methodologies and from human clinical studies. To our knowledge, paroxetine and agomelatine have not been explored in this field until now.

DA has multiple effects that promote sexual behavior by stimulating the copulatory capacity and genital reflexes. In the nigrostriatal pathway, DA influences motor activity; in the mesolimbic pathway, DA activates motivated behavior, including copulation; and in the medial preoptic area, DA controls genital reflexes, copulation patterns, and sexual motivation [31].

### 4.1. Paroxetine Reduces TH Immunoreactivity in All Meso-Diencephalic Dopaminergic Systems

Treatment with paroxetine frequently causes sexual dysfunction in humans for short, medium, and long durations [32], and this adverse effect is related to the hypofunction of the dopaminergic system in nigrostriatal and mesolimbic/mesocortical pathways, as reported in various publications [18,31,33,34]. Similar results have been observed after the administration of fluoxetine or escitalopram, which induce a decrease in DA neuron firing rate in the VTA [35]. It is suggested that this class of antidepressant acts through 5-HT2C receptors [17]. Recently, Demireva et al. (2018) [36] have demonstrated that SSRI-induced motor deficits in mice can be reversed by systemic or SNr-localized 5-HT2C receptor antagonism. SSRIs induce SNr hyperactivity and SNc (dopaminergic) hypoactivity that can also be reversed by systemic 5-HT2C receptor antagonism. Considering the critical role of DA in hedonic processes, the decrease in firing activity by SSRIs might contribute to the resistance to antidepressants in some patients. 

Our results additionally show a general decrease in TH immunoreactivity in these dopaminergic systems after treatment with paroxetine, which is consistent with the results of the authors cited above, and which could result in a reduction in motivated behavior, including copulation (mesolimbic pathway) and overall sexual dysfunction.

We also showed a previously unreported reduction of TH reactivity in DA neurons of the ZI. These neurons originate the incerto-hypothalamic tract, which innervates the anterior hypothalamus and the dorsomedial and paraventricular nuclei, and which is thought to have a stimulatory role in the release of LH [37]. More recently, other authors have proposed that ZI dopamine stimulates the release of LH and prolactin acting through glutamatergic NMDA receptors [38]. The incerto-hypothalamic pathway is involved in coordination of genital reflexes necessary for erection [39]. Therefore, the important decrease in the intensity of TH staining in ZI neurons of paroxetine-treated rats, must correspond to a decrease in the available DA and its stimulatory effect on the release of LH and sexual behavior.

Our study also shows that TH immunoreactivity is weak in the tuberoinfundibular dopaminergic neurons, and that labeling disappears from the median eminence dopaminergic axons after treatment with paroxetine. These observations are consistent with data from Lyons et al. (2016) [40], which showed that fluoxetine and sertraline, directly suppress tuberoinfundibular dopamine (TIDA) neuron activity. The hypo-function of this dopaminergic inhibitory system will be accompanied by hyperprolactinemia, as in treatment with other antidepressants or antipsychotics. Among the consequences of hyperprolactinemia in men are erectile dysfunction, with reduced sexual desire, and sometimes ejaculatory and orgasmic disorder [41,42,43]. 

Our findings of decreased immunoreactivity to TH after treatment with paroxetine are consistent with other published data reporting a reduction of TH gene expression in VTA and SN areas after fluoxetine administration for 16 and 31 days [44], or a decrease in TH mRNA in the locus ceruleus after chronic paroxetine administration [45].

The sexual dysfunction linked to antidepressant treatment has also been studied in humans via neuroimaging, showing that paroxetine and other SSRIs reduce the activity of brain networks involved in processing the motivational and emotional aspects of sexual function [46,47]. 

To summarize, many clinical and experimental studies show that SSRI antidepressants (including paroxetine) can alter all phases of sexual activity, from desire to arousal, orgasm, and ejaculation. Sexual dysfunction in males results in the inability to achieve erection or reach orgasm, while in women the problem is usually a decrease in sexual desire and delay or difficulty in reaching orgasm. In addition, there is growing experimental evidence that inhibition of meso-diencephalic dopaminergic systems is a determining factor in the aforementioned effects [18].

Our research shows that treatment with paroxetine reduces TH labeling in the incerto-hypothalamic and tuberoinfundibular dopaminergic systems. Hypo-function in these systems probably leads to a decrease in hypothalamic-pituitary-gonadal axis activity, which has been shown in clinical studies after treatment with antidepressants [48,49]. 

### 4.2. Agomelatine Treatment Also Slightly Reduces Dopaminergic Activity but Less Than Paroxetine

This study also shows that treatment with agomelatine for 28 days reduces immunoreactivity to TH in the SNc, although the effect is less intense than after treatment with paroxetine. Moreover, our data show no difference in immunoreactivity for TH in the VTA between control rats and those treated with agomelatine, which suggests that agomelatine does not affect the activity of the SNc and VTA dopaminergic neurons in the same way.

Agomelatine has an antidopaminergic action similar to melatonin [50], although the decrease in immunoreactivity to TH produced by agomelatine is not as intense as that produced by paroxetine. However, agomelatine increases levels of DA and NA in the frontal cortex (via mesocortical) by 5HT2C receptor blockade, but it does not affect the DA in the striatum and accumbens (nigrostriatal and mesolimbic pathways) [4,51]. Our data shows moderate reactivity of dopaminergic axons in the striatum as well as an intense TH labeling in the mesocortical fibers (not shown), which is consistent with previously published results.

It has also been reported that agomelatine stimulates tuberoinfundibular dopaminergic neurons, thereby inhibiting the lactotrope cell activity [52]. We found no difference in TH staining intensity in the ZI and in the tuberoinfundibular dopaminergic system between the control rats and those treated with agomelatine. Thus, we cannot confirm or reject this assertion.

In summary, treatment with agomelatine has a moderate inhibitory effect on the dopaminergic nigrostriatal system, but its action on the meso-limbic and meso-cortical pathways is barely noticeable and is much lower than that produced by paroxetine administration. Additionally, agomelatine does not seem to inhibit the incerto-hypothalamic and tuberoinfundibular dopaminergic systems.

These data are consistent with previous observations that show notable differences in the impact of various antidepressants on the dopaminergic system. The differential effects of paroxetine and agomelatine on the TH immunoreactivity and dopaminergic systems may partly explain the impact that the tested treatments have on sexual function, including the high frequency of sexual dysfunction in paroxetine-treated patients.

The observations on sexual behavior were negative and no mating behavior was observed. These negative results could be due to some limitations in the experimental design, even though the rat was ovulating and sexually receptive during the contact with the male. The lack of mating behavior could be due to the sexual encounter that took place in a new cage and not in the female’s usual cage. On the other hand, the observation took place at 18:00 h, which was the same time of usual contact with the observer who had previously administered the drugs. Additionally, the observation period of 5 min was perhaps scarce, and the observation method could have been different, for example using a recording without the presence of the observer. 

## 5. Limitations.

The ImageJ data could only be used for statistical analysis on the substantia nigra and the VTA since these areas are nuclei in which many neurons are observed in each microscopic cut. However, this method could not be applied to the other brain territories analyzed because the neuronal population is much smaller and the representativeness of the statistical analysis in the cuttings could not be guaranteed.

The presence of negative results in the observation of sexual behavior could be due to some methodological limitations of the design that should be taken into account for future studies in order to reproduce suitable results in this field.
